# Effects of Botanical Ingredients Addition on the Bioactive Compounds and Quality of Non-Alcoholic and Craft Beer

**DOI:** 10.3390/plants11151958

**Published:** 2022-07-28

**Authors:** Andrei Borșa, Mircea Valentin Muntean, Liana Claudia Salanță, Maria Tofană, Sonia Ancuța Socaci, Elena Mudura, Anamaria Pop, Carmen Rodica Pop

**Affiliations:** 1 Department of Food Engineering, Faculty of Food Science and Technology, University of Agricultural Sciences and Veterinary Medicine Cluj-Napoca, 400372 Cluj-Napoca, Romania; andrei.borsa@usamvcluj.ro (A.B.); elena.mudura@usamvcluj.ro (E.M.); anamaria.pop@usamvcluj.ro (A.P.); 2 Department of Technical and Soil Science, Faculty of Agriculture, University of Agricultural Sciences and Veterinary Medicine Cluj-Napoca, 400372 Cluj-Napoca, Romania; mircea.muntean@usamvcluj.ro; 3 Department of Food Science, Faculty of Food Science and Technology, University of Agricultural Sciences and Veterinary Medicine Cluj-Napoca, 400372 Cluj-Napoca, Romania; maria.tofana@usamvcluj.ro (M.T.); carmen-rodica.pop@usamvcluj.ro (C.R.P.)

**Keywords:** natural antioxidant, non-alcoholic beer, low-alcohol beers, craft beer, sensorial profile

## Abstract

Special beers, known as artisanal, are progressively gaining consumer preference, opening up competition, and acquiring more space in the market. Considering that, exploration for new formulations is justified and plants represent a source of novel compounds with promising antioxidant activity for this beer segment. This paper aims to evaluate the current knowledge on the role of botanical ingredients on the final yield of bioactive compounds in special beer, and how these molecules generally affect the sensory profile. Furthermore, the estimated difficulties of implementation, taking into account the new processes and the relative cost, are discussed. The addition of plants to beer could serve the interests of both the industry and consumers, on one hand, by improving the functional properties and offering a unique flavor, and on the other hand by adding variety to the craft beer landscape. This paper provides guidance and future directions for the development of new products to boost the brewing industry. Brewing processes might affect the valuable compounds, especially the phenolic content. Consequently, future studies need to identify new methods for protecting the level of bioactive compounds in special beer and increasing the bio-accessibility, along with optimization of the sensory and technological properties.

## 1. Introduction

Beer is an alcoholic beverage frequently consumed globally. In Europe, the average beer consumption in 2020 was 65 L per capita [1]. The nutritional interest in beer has increased in recent years due to antioxidant compound content and low ethanol levels. Most of the valuable compounds in beer (amino acids, vitamins, minerals, and phenolic compounds), with a role in the human diet, come from basic raw materials [2]. The conventional ingredients are malted cereals, water, yeast strains, and hops, in various proportions. Brewing handicraft evolved from ancient brewers and the German purity law to high-scale, modern industrial brewing [3]. Assortment diversification has been influenced by several factors, such as the growing interest in craft beer; consumer concern for a healthy diet; the economic rationale from producers; the development of gluten-free brewing; and consumer interest in unique sensorial products [4]. Over the years, different types of beer have been developed in various countries, due to the variety of raw materials and technologies, making beer a subject of constant research. The wide range of raw materials (fruits, wine, honey, plants, or adjuncts) which can be used in the production of beers provides opportunities for brewers to conquer new markets and meet the demands of consumers [5,6,7]. Beer production is a complex technological process that combines physical, chemical, and biological (microbiological and biochemical) processes. Modeling and optimizing the parameters of each stage will ensure the quality of the finished drink [8]. Millennial beer drinkers are getting more and more sophisticated, seeking products that are best suited to their meal type, health impact, and specific occasions. In recentyears in Europe, harmful drinking patterns are declining and the trend of consumption is following the lower strength products. In that sense, brewers have to keep up with the consumers’ wishes and expand the assortment of beer [9]. Given this, the market for special beer: non-alcoholic beer (NAB); low alcoholic beer (LAB); and Craft beer (CB) with improved functionality, new flavors, and olfactory and visual stimuli has significantly increased [10,11]. These products have a unique sensorial profile and significantly higher content of natural antioxidants, particularly phenolic compounds. Due to the storage time, the antioxidant potential in beer declines, hence, attention has now shifted toward increasing the antioxidant activity of beer by the addition of natural compounds for mitigating the effect of free radicals [12].

Craft brewing is becoming a global mainstream trend and consumers focus more and more on gaining new sensations (trade up quantity for higher quality) and reducing their alcohol intake, thus generating opportunities for innovations in functional craft beers, the use of new ingredients, and elevating perceptions of the product. This review attempts to analyze the possibilities of increasing the functional properties of special beer by the addition of various plant materials that are a rich source of bioactive compounds and to evaluate their impact on the sensory profile.

## 2. An Overview of Artisanal Beers

### 2.1. Bioactive Compounds and Nutritional Composition

The brewing process starts with malting, when the cereals are germinated under controlled conditions, and continues with mashing, whose final compound is wort, a mixture rich in sugar compounds. Wort is subsequently boiled with hops, resulting in a liquid with a dense foam due to protein precipitation. After it cools to a temperature that is compatible with yeast, it is oxygenated, and the fermentation process begins. The final stage is maturation, where the beer is stored for several weeks and then packed. In industrial settings, between maturation and packaging there is a filtration process; crafted beers are usually unfiltered or ice-cold separation is used [13]. In the production of NABs, dealcoholizing is performed during fermentation by biological methods (modifying yeast fermentation or strains) or physical methods applied after fermentation (thermal or membrane processes) [14].

The high demand for a variety of products encourages beer crafters to produce innovative drinks by maintaining the four ingredients of beer while varying the malted cereals blend, the yeast strains or the hops but also by adding fruit, vegetables, plant extracts or aging the beer in wooden barrels (previously used for wine or spirit aging) to modify beer sensory characteristics. CB masters produce LAB, NAB, and gluten-free craft beer to respond to people’s needs and to make beer appealing to women, teenagers, and people with gluten intolerance [15]. LAB with sodium addition may be suitable for athletes, acting like isotonic drinks, to reduce post-exercise fluid losses [16]. The main difference between craft beers and industrial ones are the varieties of malts, hops, and yeasts used in the production process and batch dimension, which allows crafters to experiment with ingredients with a focus on aromatic notes given by raw materials or original additions [17].

CB consumers are usually young adults (20–39 years) with high–medium incomes, who look for new tastes, and appreciate small, non-industrial breweries and new experiences [18]. Aquilani et al., 2015, investigated consumer preference for CB and the probability of beer drinkers choosing CB was influenced by the beer’s attributes (aroma, foam, carbonation) and their consumption habits. CB was chosen for its selection of flavors (which contributed to a perception of higher quality): malted barley, chestnut, and honey [19]. Merlino et al., 2020, interviewed CB consumers during a beer festival to find out if the packaging (canned or bottled) affects the perception of a set of attributes defining beer quality: alcohol content, aroma, bitterness, body, clarity, color, sweetness, taste intensity, and turbidity. The results revealed that the two groups (traditional bottled beer consumers and innovative canned beer consumers) did not differ significantly regarding sensory preferences and could evaluate and appreciate the unique characteristics of crafted beer [20].

Besides alcohol and sugar content, beer has the potential to provide a significant amount of nutrients such as vitamins and minerals, and is a potential source of vegan B12. It is isotonic and its main ingredients have potential health benefits [21]. Protein and amino acid content are influenced by production processes: lager, ale and wheat beer usually lack protein, whereas other types of beers, regardless of their alcohol content, have a protein content of 0.3–0.6 g/100 mL. Carbohydrates contribute to the energetic value of beers, with NAB having higher concentrations than regular beers (4.5–14 g/100 mL vs. 3.3–4.4 g/100 mL). The addition of fruits increases the concentration of bioactive compounds such as flavonoids, tocopherols, ascorbic acid, and carotenoids, improving beer quality and impact on consumer health [10]. Beer is rich in antioxidant compounds such as phenolic compounds and melanoidins generated mostly by barley and hops varieties used in beer recipes [2].

During the brewing process, phenolic compounds are exposed to qualitative and quantitative changes. At the end of the brewing process, 60% of the phenolic content of malt is lost because of high-temperature pasteurization and filtration in large industrial-produced beer, while craft beer, which is usually unfiltered, has a higher concentration of phenolic compounds. Due to the production processes, NAB and LAB have lower concentrations of phenolic compounds [22]. Silva et al., 2021, identified and quantified phenolic compounds in 14 samples of Brazilian craft beers by HPLC-DAD (high-performance liquid chromatography with diode array detection) with the aim of finding a simple method to analyze beverages or other matrices. Rutin and formononetin were present in all samples; catechin, epicatechin, and caffeic acid presented the highest concentrations, influenced by the addition of coffee, vanilla, or honey in the CB brewing process [11]. Phenolic acids are considered a valuable source of antioxidants and also have a role in the sensory properties, color, and stability of beer flavor and the scavenging activity of free radicals, and their presence in CB, is a benefit for the consumer. Marques et al., 2017, characterized CB and its bioactive compounds, focusing on phenolic compounds. Gallic acid, caffeic acid, ferulic acid, and p-coumaric acid were found in four different types of CB, with caffeic acid showing the highest concentration [23]. Silva et al., 2021, reviewed the potential biological activities of craft beer and concluded that phenolic compounds, with their antioxidant activity, may contribute to a risk reduction of cancer and cardiovascular events if moderately consumed (1 drink/day for women, 2 drinks/day for men). Beer is also a natural source of silicon, contributing to a reduction in neurodegenerative disease development by decreasing aluminum bioavailability and inhibiting bone resorption in postmenopausal women with osteoporosis [24]. Polyphenols, prenylflavonoids, beer color, haze stability, and protein in beer are factors that characterize special beer (CB, Lab, and NAB) quality and stability. Polyphenols contribute to flavor, bitterness, foam, color, and colloidal stability, while prenylflavonoids ensure foam stability and present antioxidant activity, prolonged microbial activity and flavor. Beer color changes during storage as oxidative processes take place. Catechins and epicatechins are involved in haze formation and colloidal stability, but also contribute to bitterness, and amino acids from malt provide free amino nitrogen (FAN) which is responsible for the formation of flavor compounds [9]. Cheiran et al., 2019, evaluated phenolic and nitrogenous compounds found in three types of CB by HPLC−DAD−ESI−MS/MS (high-performance liquid chromatography with a diode array detector coupled to a mass spectrometer by an electrospray ionization source) and identified 57 different phenols (12 newly described in beer) and 11 nitrogenous compounds [25].

Spent hops are a rich source of proteins, polyphenols, and essential oils [26], and could be used in the artisanal beer segment for new flavors and potential health benefits. Spent hop extracts have estrogenic effects by two mechanisms: as aromatase inhibitors that could lower the risk of estrogenic carcinogenesis and as estrogen receptor inhibitors which may help manage menopausal symptoms. Its constituents are involved in detoxification and inflammatory pathways and in cellular apoptosis and also have selective inhibitory activity upon some of the CYP450 enzymes: 1A1, 1A2, 1B1, 2C8, 2C9, and 2C19, which may lead to drug interactions [27]. Yeasts used during the fermentation process may be a source of probiotics. Use of *Saccharomyces cerevisiae* var. *boulardii* instead of *S. cerevisiae* showed increased antioxidant activity, lower alcohol content, similar sensory attributes, and higher yeast stability, resulting in a beer with increased health benefits [28].

### 2.2. Sensorial Assessments and Consumers’ Perception

In beer evaluation, different sensory analysis methods are used: difference tests, descriptive tests, ranking tests (such as the nine-point hedonic scale), the free-choice test, the drinkability test, or a mixture of two or more methods. Trained or untrained panelists taste different types of beers under the same conditions and rate it using different ranks or scales to see whether there are differences between samples, whether some characteristics are more intense than others, andwhether it is more drinkable or preferred. Ideal conditions are an ambient temperature (21 °C), glass receptacles to serve the tested beer (which should be cooled to around 4 °C), no additional smells or noises in the room, three digit codification of each sample, and trained panelists who are aware of the beers’ ingredients and can appreciate the raw materials’ contribution to a beer’s taste [29].

During brewing, volatile and phenolic compounds are formed and contribute to the beer’s identity, influencing taste and aroma. Ethyl acetate, ethyl caproate, ethyl caprylate, isoamyl acetate, isobutyl acetate, phenylethyl acetate, and ethyl octanoate are esters that contribute to beer aroma: buttery-like, sour-apple-like, fruity, flowery, etc. Phenolic compounds may add an astringent or bitter flavor and modify the beer sensory profile [30]. Beer aging is a very important step for the final product and chemical reactions such as the Maillard reaction, Strecker degradation of amino acids, and oxidation of unsaturated fatty acids take place and change its sensory profile. Chemical and sensory aging indicators are used to evaluate differences between fresh and aged beer but have the disadvantage of being subjective and generic. Chemometrics analysis manages to solve this disadvantage, correlating several dependent variables with one or more independent variables in a multivariate analysis procedure to better understand beer aging variables and the influences of raw materials, process, and storage conditions on flavor [31]. The most used descriptive test used by beer crafters to identify the flavors and assess their intensity is Quantitative Descriptive Analysis (QDA). Different scales are used and tasters evaluate the presence of a flavor (standardized by the Beer Flavor Wheel) and its intensity on the chosen scale, which is not standardized and may be a source of bias because giving numbers to intensities is subjective [32]. Such a scale is the nine-point hedonic scale which evaluates consumer preference for a product. Trained or untrained potential customers taste the beer specialties and assess on a scale from 1 (dislike extremely) to 9 (like extremely) their preference for some of the aspects of the product (taste, appearance, flavor, etc.).

Preferences among alike products, cross-cultural use, and the multitude of products to be ranked are disadvantages of using this tool. Associating *R*-index analysis or two-stage ranking based on words, not numbers, could improve the hedonic scale’s statistical significance [33]. Despite the potential problems, the nine-point hedonic scale is largely used by CB or NAB producers for sensory analysis and evaluating the consumer opinion of the product. Tozetto et al., 2019, made a sensorial evaluation of a pilsner-type ginger craft beer with 85 untrained testers using the nine-point hedonic scale for color, taste, aroma, bitterness, and appearance evaluation and a five-point scale for purchase intent assessment [34]. Mazengia et al., 2021, evaluated a lager-type Moringa stenopetala leaf extract (LEMS) crafted beer with 15 well-trained panelists using the nine-point hedonic scale to assess the effect of LEMS and storage time on the color, foam stability, bitterness, mouth feel, aroma, flavor, and overall acceptability of the beer [12]. Ale beer brewed with rice and fruit by-products was sensory evaluated by Sriwichai et al. using 20 volunteer panelists usinga linear scale followed by the nine-point hedonic scale. Odor (malty-like, hop-like, caramel-like, yeasty-like), taste (sourness, sweetness, bitterness), appearance (turbidity), and overall appreciation were assessed. Similar appreciation with a control beer showed that functional beer with improved phenolic content and low-alcohol content can be produced from paddy rice, rice berry powder, banana-peel, and coffee pulp [35]. The addition of rice flakes and soursop pulp to artisanal beer was sensory evaluated on the nine-point hedonic scale; color, bitterness, aroma, taste, and overall impression were assessed by 100 untrained persons and it was concluded that this addition was a viable alternative which was well accepted by tasters [36].

The nine-point hedonic scale is associated with chemometric tools, a multivariate approach to data analysis. Ghasemi-Varnamkhasti et al. evaluated nine aftertaste sensory attributes of seven commercial NABs: bitter, sour, sweet, fruity, licorice, artificial, body, intensity, and duration using. The beers were coded and randomly served to panel members who scored the attributes from 0 to 9 (poor to excellent). The neural network method showed promising results in predicting beer brands, but the radial basis functions (RBS) method showed the best accuracy in beer classification [37]. Medoro et al., 2016, compared the sensory profile of five Italian craft beers from a beer taster expert’s point of view and by sensory methods; 12 judges were trained for the sensory analysis and evaluated 28 beer attributes (odor, visual, gustatory, and textural) while the beer sommelier characterized each beer according to his own experience. The sensory methods were comparable to the description made by the beer expert with the advantage of offering sensory profiles, which could help crafters to target specific segments of the beer market [38].

NAB is usually considered bland and watery compared to alcoholic beer but it is appreciated by consumers who are interested in having a healthy lifestyle. Sensory analysis showed that flavor and aroma contribute to the likeliness of consuming NAB and can represent a starting point for creating innovative NABs [39]. Flavor defects in NAB and LAB are due to eliminating or reducing ethanol content processes in beer production. Volatile compounds such as higher alcohols and esters are lost during ethanol removal or thermal processes and contribute to immature flavor and inharmonious taste. Limited fermentation does not allow carbonyl reduction, resulting in an unpleasant. Bitterness, foam stability, and microbial stability are also negatively affected by alcohol removal [40]. Muller et al., 2021, evaluated 23 types of NAB and made a sensory analysis of flavor characteristics (fruity, aromatic, fragrant, cereal, sweet, malty), fullness, harmony, carbonation, and bitterness and built a specific sensory scheme after identifying the volatile compounds responsible for an NAB’s aromatic profile. Training of the panelists was important to use the sensory analysis as a tool for NAB crafters [41]. In the study conducted by Lafontaine et al., 2020, the chemical and sensory profiles of 42 different NABs were evaluated. The results revealed the preferences of consumers for beers with citrusy, tropical, and stone fruit aromas. Botanical additions can have a significant impact on the resulting aroma and flavor of these products [42].

## 3. Strengths and Weaknesses of Botanical Addition

Functional beers are a class of non-traditional beers, with functional ingredients added during or after the traditional brewing process, which contain bioactive compounds that can bring a health benefit to the consumer. To evaluate this potential benefit, total phenolic content, phenolic profile, antioxidant activity, and volatile profile are usually tested. Sensory acceptability is further evaluated to predict consumers’ choice and willingness to drink functional beers [43]. Djordjevic et al., 2016, added Melissae folium, Thymi herba, Juniperi fructus, Urticae radix, and Lupuli strobuli extracts to a commercial lager beer type “with the aim of obtaining a beer with increased functional and new sensory features”. The content of phenolic compounds, antioxidant activity, and the sensory acceptability were evaluated. The thyme addition contributed to the highest polyphenols content (1301.54 ± 5.23 mg GAE/L), but it was not taste-appealing for the consumers, while lemon balm extract, as expected, was the most appreciated for its aroma and had an important polyphenol content (1112.3 ± 4.52 mg GAE/L) [44]. Fruits may be added to beer to improve bioactive compounds composition and antioxidant activity. Cherry, raspberry, peach, apricot, grape, plum, orange, and apple added to the fermentation process led to higher polyphenols and flavonoids content and higher antioxidant activity than conventional beers, with cherry beer exhibiting the highest values. Catechin, quercetin, myricetin, and resveratrol were identified in most of the fruit beers [5]. Fruit waste can be a source of beer aromatizer. Ricci et al., 2019, added lacto-fermented apple pomace to cream ale beer and observed an improved volatile profile of the beer which must be further sensory evaluated to find an acceptable proportion of fruit waste addition to satisfy consumer expectations [45]. Suthovski et al., 2021, added pecan nutshell aqueous extract (PSAE) to an ale crafted beer with medium alcohol volume and low sweet taste, characteristic of the oatmeal stout style. Despite PSAE’s antioxidant capacity, its addition did not increase the crafted beer’s antioxidant activity, however, it was well accepted by consumers and further studies should improve PSAE incorporation into craft a beer with a higher antioxidant capacity and value of the nut by-product [46]. The effects of adding walnut, chestnut, cocoa, green tea, coffee, honey, or licorice during the fermentation process were investigated by Nardini et al., 2020, and compared with conventional beers. Total phenolic content, total flavonoid content, and antioxidant capacity were higher than in conventional beer. Most were enriched in catechin, epicatechin, rutin, myricetin, quercetin, and resveratrol which led to an increase in the nutritional quality of beer [47].

The results of phenolic compound content for the beers developed are shown in Table 1. The studies reviewed have highlighted the transfer of bioactive substances from the botanical ingredients to the final beer and showed that the increase in polyphenol content depends proportionally on the concentration of these in beers. The use of plants in the maturation stage or fermentation step showed positive results with higher content of total phenolic (TPC) compared with the boiling stage.

Bustos et al., 2019 [48], reported higher values of TPC for a porter-style craft beer enriched with *Parastrephia lucida*, compared with the control beer. However, an increase in TPC can be achieved regardless of the stage of addition. The results presented by Pereira et al., 2020 [49], showed that the increase in polyphenol content depends proportionally on cashew peduncle and orange peel concentration in beers. Furthermore, the sensory evaluation revealed a preference for the F6 sample (which contained 10% of cashew peduncle and 0.6% of orange peel).

Table 2 shows the data for antioxidant activity of the new beers and the antioxidant tests used: DPPH, FRAP, ABTS, and ORAC. The results presented in Table 2 are in line with the findings for the total phenolics; an increase in beer’s antioxidant activity occurs after the addition of botanical ingredients. An exception was for the lager beer with the addition of *Melissae folium*, *Thymi herba*, *Juniperi fructus*, *Urticae radix,* and *Lupuli strobuli*, which showed low increases in antioxidant activity. In a study conducted by Piva et al., 2021 [60], the use of the extract of *Ocimum selloi* in craft beer after the fermentation step improved the antioxidant capacity, measured by DPPH radical scavenging activity, from 30.09% to 69.9%.

Table 1 and Table 2 summarize the effects of plants’ addition to different types of beer during stages of the brewing process upon bioactive compounds found in beers. Studies concerning the bioavailability, metabolization, and the absorption of phenolic compounds, must be included in future studies of the special beer segment. A gap in the papers reviewed points to the sensory analysis of the developed beverages, which is not unanimous in the literature, and this approach is an essential tool to evaluate the acceptance by consumers.

Plants’ phytochemical compositions depend on several factors, such as the genotype, climatic conditions, stage of ripeness when incorporated, preparation methods such as extraction, degree of crushing, etc. [63,64,65]. Therefore, to produce beers with a high biological value, it is highly desirable to identify new methods for protecting or even increasing the level of health-related compounds in beer.

At the lab scale, adjuncts are generally successfully added in every step of the brewing stages however, although they can initially induce a high intake of antioxidant compounds into the mix, the latter can be destroyed during the process. As previously researched, mashing, boiling, and wort clarification or filtration have been shown to reduce their quantities. The antioxidant capacity can decrease even more from fermentation to bottling [66,67]. Supplementary interaction between polyphenols, proteins, and polysaccharides due to the new complex combination of ingredients can induce a colloidal haze [56,68].

When analyzing the final product, as an NAB or LAB, each of their final characteristics is important: alcohol content shows the brewing efficiency and product classification, thus the higher amount of fermentable sugars must be taken into account; significant polyphenolic composition can be considered a selling point (marketing) and needs to be considered as a quality indicator since the different ingredient combinations and wort-production technology influence the taste, color, foam, beer turbidity, bitterness, colloidal, and sensory properties as well as the stability and shelf-life of beer. Therefore, Table 3 presents the main specifics of the different plant adjuncts researched in the craft beer domain, with an estimated difficulty of implementation taking into account the new processes and the relative cost related to the additional time needed to create the final product and the capital needed for production [11,22,69,70].

The main technical characteristics of plant addition, which differ from the classical brewing proces, are shown in Table 3. Their specific antioxidant compound share and sensory enhancements, which could in future be considered their main selling points, are also presented, together with the disadvantages which need to be controlled or disguised, as the case may be for each assortment. By combining these factors, we can estimate the difficulty of implementation at a production scale as well as the cost required to purchase additional equipment and the additional time needed for the creation of medium-sized monthly batches. The implementation cost includes, for example, the hydro-distillation or ethanol extraction process and equipment (which may need to be bespoke and, therefore, more expensive).

General positive aspects observed: improved antimicrobial and antioxidative properties due to lower pH value and extract content; longer shelf life; more stable flavor and aroma; increased foam smoothness and stability; health benefits generated by the antioxidants that attract consumers based on their perception of disease prevention through a functional diet; greater concentration of volatiles with fruity, floral, and sweet aromas preferred by the consumers.

General negative aspects observed that need to be taken care of in order to introduce the product to the market: adjunct addition in the maturation step induced low increases in antioxidant and phenolic content of enriched beers but in some cases improved the appropriate techniques needed to be mastered to obtain an acceptable quality product; new processes must be added that do not negatively influence the microbiological load, such as crushing and homogenizing the plants before adding them to beer to increase the area of contact with the surrounding beer and the breakage of plant cell walls, in releasing phenolic compounds into the solution; increased viscosity and higher yeast suspension increased the beers’ turbidity, foam smoothness, and stability; excessive foaming due to the complexity of the reactions between the ingredients; antioxidant losses throughout the entire brewing process; beer clarification processes using clarifying agents, such as carrageenan, silica gel, or polyvinylpolypyrrolidone (PVPP), should be applied to obtain colloidal stability; lack of specific research on LAB/NAB specific optimization processes.

Although the essential oils from different hop species have similar classes of chemical compounds and levels of complexity, they have very different effects on the sensory characteristics of all kinds of beer [71], generating a variety of flavors and aromas that are described as “floral,” “fruity,” “spicy,” “herbal”, etc. Riu-Aumatell et al., 2014 [72], evaluated the volatile profiles of LAB/NAB and alcoholic beers and found that the latter had higher levels of fermentation compounds such as esters (isoamyl acetate, ethyl hexanoate, ethyl octanoate), and alcohols (1-octanol, decanol, isobutanol, isoamyl alcohol). Beer’s qualitative attributes are initially compromised by conventional pasteurization, which the brewing industry uses to ensure microbiological stability. Afterward, dealcoholization through physical processes may be to blame for their even lower level in LAB, or their generation may be constrained by biological methods. Pasteurization is even more important in botanical ingredients addition since it increases biological activity, and the stability of the final product must be ensured. It can be avoided for example, by replacing it with a cold-sterilization based on filtration through silica microparticles (5, 10, 25 or 50 μm), functionalized with essential oil components, as shown by Nataly Peña-Gómez et al., 2020 [73]. This technology using naturally occurring antimicrobial molecules on supports still needs to be optimized and will increase the investment and production costs but increase the quality of the beers. As an alternative, one can accept the reduction in aroma compounds due to thermal treatment and compensate for it by using essential oil extracts rich in bioactive compounds. The latter can be introduced especially during fermentation (where there is no longer a thermal process) to cover the lack of flavor and to add new aroma profiles to the liking of contemporary customers. By using them in conjunction with different extracts, such as propolis or *Junupei fructus*, improved antimicrobial and antioxidant properties prolong shelf life.

Practically, only imagination is the limit of LAB/NAB craft brewers. Since the perception of volatiles varies with their concentrations, combinations, and threshold levels in different beer matrices or model systems, the latter can, utilizing current research, penetrate new markets with new sensational brews simply by testing them out.

## 4. Conclusions

Brewers are sourcing innovative solutions to obtaining products of higher quality that meet the consumer’s demands. In the literature, few articles comprehensively study artisanal beers with botanical incorporation in different phases of the manufacturing process and how these constituents influence the attributes and sensorial characteristics of the final product. Therefore, this paper highlights the promising potential for the development and use of botanical ingredients as an additional source of bioactive compounds and increased antioxidant activity in the special beer segment. Plants could be used as an adjunct to special beer, to improve the stability, and shelf-life, and to create an authentic product with a complex and rich aroma. This paper provides guidance and future directions for the development of new products to boost the brewing industry. The utilization of botanical ingredients could represent the next step in special beer’s development by giving breweries strategies for a lineup of distinctive products. Brewing processes, such as mashing, boiling, fermentation, maturation, and storage might affect the valuable compounds, especially the phenolic content. Consequently, future studies need to identify new methods for protecting the level of bioactive compounds in special beer and increasing the bio-accessibility, along with optimization of the sensory and technological properties.

## Figures and Tables

**Table 1:** Studies regarding the production of special beer and their total phenolic (TPC) and total flavonoid content (TFC).

Plant Added	**Addition** Part	Type of Beer	Brewing Process Stage	Conventional Beer TPC (mg GAE/L)	Enriched Beer TPC (mg GAE/L)	Conventional beer TFC (mg QE/L)	Enriched Beer TFC (mg QE/L)	References
*Parastrephia lucida* (romero or tola)	dried leaves	American Porter	maturation	413.21	480.16–800.64	333.47	346.67–601.12	[48]
*Citrus sinensis* (orange)	peel	Craft	fermentation	516.4	515.9–726.6	-	-	[49]
*Anacardium occidentale* (cashew nut)	peduncule				640.9–722.3	-	-	
Propolis	extract	Golden ale	maturation	242.0	253–306.5	16.9	19.6–26.9	[50]
Prokupac grape	fruit	Craft	fermentation	467.78	550–569.3	-	-	[51]
White grape	pomace	Lager	fermentation	219.028	321.584–501.459	-	-	[52]
*Chenopodium quinoa* (quinoa)	seeds	Non-alcoholic	wort boiling	208.66	221.64–271.24	20.65	24.29–30.85	[53]
*Lycium barbarum* (goji berry)	fruit	Amber ale	fermentation	335	357–623	-	-	[54]
*Hibiscus sabdariffa* (red sorrel)	calices powdered extract	Ale	maturation	294.18	410.62–743.16	-	-	[55]
*Solanum melongena* L. (eggplant)	peel extract	Lager	after maturation	0.416–0.426	0.433–0.631	0.063–0.065	0.074–0.175	[56]
*Mangifera Indica* (mango)	fruit/pulp	Craft	fermentation	187.4	218.6–267.6	-	-	[57]
*Cydonia oblonga* (quince)	fruit	Amber ale	maturation	13.47	15.9–17.55	-	-	[58]
*Ipomoea batatas* (sweet potato)	root	Pale ale	wort boiling	210.92	218.38–230.5	17.53	16.02–21.31	[13]
*Crataegus punctata* (dotted hawthorn)	fruit and juice	American Saison	fermentation	200	279.6–410.1	-	-	[59]
*Ocimum selloi* (green pepper basil)	leaves	Craft	fermentation	291.2	359–371.9	-	-	[60]
*Melissae folium* (lemon balm leaves) *Thymi vulgaris herba* (wild thyme) *Juniperi fructus* (juniper cone) *Urticae radix* (nettle) *Lupuli strobuli* (hops strobilus)	extract	Lager	after packaging	280.26	316–384	-	-	[44]
*Camellia sinensis* L. (tea tree)	Extract microencapsulate	Pilsner beer	after packaging	<300	<600 <1200	-	-	[61]

“-” not determined.

**Table 2:** Comparison of antioxidant capacity of special beer samples.

Plant Added	**Addition** Part	Brewing Process Stage	Control Beer—DPPH Assay	Enriched Beer—DPPH Assay	Control Beer—FRAP Assay	Enriched Beer—FRAP Assay	Control Beer—ABTS Assay	Enriched Beer—ABTS Assay	Control Beer—ORAC Assay	Enriched Beer—ORAC Assay	References
*Parastrephia lucida* (romero or tola)	dried leaves	maturation	-	-	1.88 mM TE	2.17–5.46 mM TE	1.15 mM TE	1.38–3.34 mM TE	7.86 mM TE	10.14–30.58 mM TE	[48]
*Citrus sinensis* (orange)	peel	fermentation	-	-	-	-	1604.7 µM/L	1580.06–1736.9 µM/L	-	-	[49]
*Anacardium occidentale* (cashew nut)	peduncle			-	-	-		1567.9–1725.1 µM/L	-	-	
Propolis	extract	maturation	0.533 mmol TE/L	0.491–0.576 mmol TE/L	1415.0 µmol TE/L	1555.0–1892.5 µmol TE/L	0.629 mmol TE/L	0.687–0.808 mmol TE/L	-	-	[50]
Prokupac grape	fruit	fermentation	0.73 mM TE	1.02–1.05 mM TE	1.28 mM TE	2.64–2.65 mM TE	-	-	-	-	[51]
White grape	pomace	fermentation	0.482 mmol TE/dm³	0.6–1.4 mmol TE/dm³	1.5 mmol TE/dm³	1.8–3.6 mmol TE/dm³	1.581 mmol TE/dm³	1.2–4.1 mmol TE/dm³	-	-	[52]
*Chenopodium quinoa* (quinoa)	seeds	wort boiling	4.52 mMol/TE	4.70–4.86 mMol/TE	0.94 mMol/TE	1.16–1.48 mMol/TE	-	-	-		[53]
*Lycium barbarum* (goji berry)	fruit	fermentation	-	-	-	-	-	-	8.87 mmol/L TE	10.03–16.84 mmol/L TE	[54]
*Hibiscus sabdariffa* (red sorrel)	calices powdered extract	maturation	-	-	-	-	3.94 mmol TE/L	5.71–6.67 mmol TE/L	-	-	[55]
*Solanum melongena* L. (eggplant)	peel extract	after maturation	0.926 mmol TE/mL	1.244–1.333 mmol TE/mL	-	-	0.086 mmol TE/mL	0.084–0.140 mmol TE/mL	-	-	[56]
*Mangifera Indica* (mango)	fruit/pulp	fermentation	1.44 mmol TE/L	1.44–2.05 mmol TE/L	1.04 mmol TE/L	1.32–1.69 mmol TE/L	0.97 mmol TE/L	1.25–1.74 mmol TE/L	-	-	[57]
*Ipomoea batatas* (sweet potato)	root	wort boiling	18.79 IC50 (μL)	16.05–18.69 IC50 (μL)	-	-	-	-	-	-	[13]
*Crataegus punctata* (dotted hawthorn)	fruit and juice	fermentation	0.352 mmol TE/L	0.443–2.175 mmol TE/L	0.512 mmol TE/L	0.869–1.35 mmol TE/L	0.936 mmol TE/L	1.356–2.041 mmol TE/L	-	-	[59]
*Ocimum selloi* (green pepper basil)	leaves	fermentation	30.09 % inhibition	34.0–69.9 % inhibition	-	-	-	-	-	-	[60]
*Melissae folium* (lemon balm leaves)	extract	after packaging	2.54 mM TE	3.05 mM TE	4.15 mM TE	4.51 mM TE	-	-	-	-	[44]
*Thymi vulgaris herba* (wild thyme)				3.72 mM TE		4.71 mM TE					
*Urticae radix* (nettle)				2.85 mM TE		4.25 mM TE					
*Junupei fructus* (juniper cone)				3.14 mM TE		4.55 mM TE					
*Lupuli strobuli* (hops strobilus)				2.83 mM TE		4.27 mM TE					
*Zea mays* L. (corn)	extracts of seed and cob	-	-	23.93 IC50 gmL^−1^	-	-	-	-	-	-	[62]

“-” not determined.

**Table 3:** Technical specific characteristics.

Plant Added	Brewing Process Stage	Type of Beer	Additional Tech Specifics	Extra Antioxidant Compounds	Sensory Enhancements	Disadvantages to Overcome	Difficulty of Implementation	Cost of Implementation Time/$$	References
*Parastrephia lucida* (romero or tola)	maturation	Dark	Added dried leaves in steps in a straining bag similar to the dry hopping process	-	light herbal flavor	increased astringency	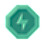	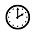 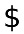	[48]
*Citrus sinensis* (orange)	fermentation	Ale	5 g/L fresh fruit peel added to beer during the first step of the fermentation process	flavonoids catechin and rutin	higher quality, more stable flavor and aroma, foam stability and longer shelf life	increased acidity, higher pH, lesser total soluble solid	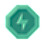	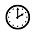 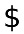	[49]
*Anacardium occidentale* (cashew nut)		-		-	-	-		-	
Propolis	maturation	-	0.25 g/L ethanolic extract of propolis by a magnetic stir bar agitation in the dark for 24 h	coumaric acid, cinnamic acid, and caffeic acid	Natural preservatives for beer.	Allergic reactions	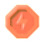	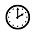 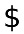 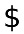	[50]
Prokupac grape	fermentation	Ale	Up to 30% of grape mash from crushed/pressed fruit	-	higher rate of fermentation, higher alcohol %	Increase of lightness of beer or shifts to redness	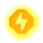	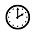	[51]
White grape	fermentation	Ale	200 g/l fresh fruits added to beer during the first step of the fermentation process	Catechin, Resveratrol	higher quality, more stable flavor and aroma, foam stability and longer shelf life	increased concentration of organic acids and decreased fermentable sugars	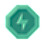	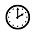	[52]
*Chenopodium quinoa* (quinoa)	wort boiling	Pils	30% quinoa wort Fermented with P. myanmarensis	Ethyl acetate, Methyl acetate and ethyl propionate	Higher antioxidant potential more volatiles, increasing the flavor	Decreased enzyme concentration, reduced ethanol content	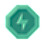	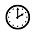	[53]
*Lycium barbarum* (goji berry)	fermentation	Ale	50 g/L ground goji 1 h boiling at 100 °C	rutin and 2-O-β-D-glucopyranosyl-L31 ascorbic acid	lower turbidity, high color intensity, caramel and coffee-like taste	Reddish color, plant odor	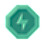	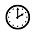 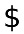	[54]
*Solanum melongena* L. (eggplant)	after maturation	Lager	10 mg/mL extract from eggplant peels	anthocyanins delphinidin-3-rutinoside, delphinidin-3-glucoside and delphinidin-3-rutinoside-5-glucoside	Good stability	reddish color because of the presence of anthocyanin pigments	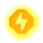	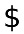 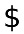	[56]
*Mangifera Indica* (mango)	fermentation	Light beer	heated homogenate of raw fruit pieces or mango leaves, followed by pasteurization at 15 PU	Maltol, 4 H-Pyran-4-one, 2,3-dihydro-3,5-dihydroxy-6-methyl, 5-Hydroxymethylfurfural, Furan-2-carboxaldehyde, 5-(1-piperidinyl)	lower pH, higher micro stability	increased risk of beer contamination, [32].	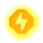	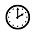 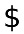 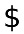	[57]
*Ipomoea batatas* (sweet potato)	wort boiling	Pale Ale/LAB	sweet potato flakes dried in the absence of light replacing 50% of grist (crushed malt) and previously held for 1 h at 62 °C for starch gelatinization	High anthocyanin and β-carotene content	balance between flavor and aroma; cheaper costs	-	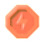	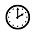 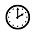 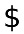 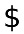	[13]
*Crataegus punctata* (dotted hawthorn)	fermentation	Ale	10% (*w*/*v*) juice/fruit in the last 3 days of fermentation	-	reduces the pH, improves the aroma compounds	Less foaminess	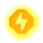	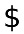	[59]
*Ocimum selloi* (green pepper basil)	fermentation	Lager/NAB	aqueous extract at 0.1% (m/v) added after the fermentation step	-	increased shelf-life performance	-	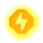	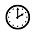 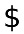	[60]
*Melissae folium* (lemon balm leaves)	after packaging	Pils	Ethanol-water extraction from leafs Ethanol-water extraction from roots Ethanol-water extracts from berries	flavonoids, rosmarinic acid and triterpenes	improved antimicrobial and antioxidative properties	-	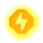 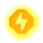 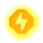 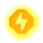 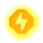	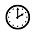 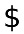 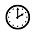 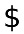	[44]
*Thymi vulgaris herba* *(wild thyme)*		Wheat beer		flavonoids and triterpenes		-			
*Urticae radix (nettle)*		Pils		lectins, sterols (β-sitosterol), lignans.		Plant/Herbal flavor			
*Junupei fructus* (juniper cone)		Lager		flavonoids, tannins, oligomeric proanthocyanidins		-			
*Lupuli strobuli* (hops strobilus)		Blond, red		flavonoids, tannins, oligomeric proanthocyanidins		-			
*Zea mays* L. (corn)	-	Chicha Morada NAB	Added Seed and cob, boiled at 100 °C for approximately 20 min; cheaper row material	Anthocyanins cyanidin-3-glucoside, pelargonidin-3-glucoside, peonidin-3-glucoside	purple color	The degradation of anthocyanins in beer is related to the thermal process.	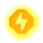	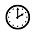	[62]

Difficulty of implementation: 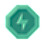 easy; 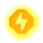 moderate; 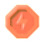 hard. Increases in the brewing time process: 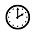 slight; 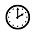

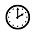
 significant. Additional cost of implementation: 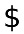 cheap; 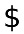

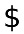
 moderate.

## Data Availability

Not applicable.
